# Dietary Betaine Supplementation Enhances Colonic Barrier Function through the Nrf2/Keap1 and TLR4-NF-κB/MAPK Signaling Pathways and Alters Colonic Microbiota in Bama Mini-Pigs

**DOI:** 10.3390/antiox12111926

**Published:** 2023-10-29

**Authors:** Liang Xiong, Kai Wang, Mingtong Song, Md. Abul Kalam Azad, Qian Zhu, Xiangfeng Kong

**Affiliations:** 1 CAS Key Laboratory of Agro-Ecological Processes in Subtropical Regions, Hunan Provincial Key Laboratory of Animal Nutritional Physiology and Metabolic Process, Institute of Subtropical Agriculture, Chinese Academy of Sciences, Changsha 410125, China; 244628426@stu.scau.edu.cn (L.X.); wkwildlife@sinogaf.cn (K.W.); yxh@stu.scau.edu.cn (M.S.); azadmak@isa.ac.cn (M.A.K.A.); zhuqian@isa.ac.cn (Q.Z.); 2 Guangdong Provincial Key Laboratory of Silviculture, Protection and Utilization, Guangdong Academy of Forestry, Guangzhou 510520, China

**Keywords:** Bama mini-pigs, betaine, colonic microbiota, immunity and inflammation, redox status

## Abstract

This study evaluated the effects of betaine supplementation in sows and/or their offspring’s diets on the redox status, immune and inflammatory levels, colonic barrier function, and colonic microbial community of offspring piglets. Thirty-six Bama mini-sows on day 3 of gestation and their weaned offspring piglets (28 d of age) were randomly allocated to the following treatments: (1) sows and their weaned offspring fed the basal diet (control group, Con group); (2) sows fed the basal diet with 3.50 kg/t betaine, and their weaned offspring fed the basal diet (sows betaine group, SB group); (3) sows fed the basal diet with 3.50 kg/t betaine, and their weaned offspring fed the basal diet with 2.50 kg/t betaine (sow-offspring betaine group, S-OB group). Six offspring piglets from each group were selected to collect plasma and colon samples on d 30, 60, and 90 after weaning. Compared with the Con group, the plasma levels of IgA, IgM, GSH-Px, and SOD during d 30–90 after weaning, IFN-α, T-AOC, and GSH on d 30 and 60 after weaning were increased, while MDA during d 30–90 after weaning was decreased in the SB and S-OB groups (*p* < 0.05). In addition, the plasma levels of IFN-γ on d 60 and T-AOC on d 30 after weaning were higher in the S-OB group than those in the Con group (*p* < 0.05). In the colon, betaine supplementation increased plasma T-AOC, GSH, and SOD levels while decreasing MDA concentration (*p* < 0.05). Betaine supplementation improved the colonic protein abundances of ZO-1, occludin, and claudin in offspring and activated the Nrf2/Keap1 signaling pathway while inhibiting the TLR4-NF-κB/MAPK signaling pathway on d 90 after weaning. The 16S rRNA sequencing results showed that betaine supplementation altered colonic microbiota composition by increasing the relative abundances of Verrucomicrobia and Actinobacteria in the SB group while decreasing proinflammatory-associated microbiota abundances (Tenericutes, *Prevotella*, and *Parabacteroides*) (*p* < 0.05). Collectively, these findings suggest that dietary betaine supplementation in sows and/or their offspring could improve offspring piglets’ redox status and immune and anti-inflammatory levels and enhance the colonic barrier function by activating Nrf2/Keap1 and inhibiting TLR4-NF-κB/MAPK signaling pathways.

## 1. Introduction

Bama mini-pigs, due to their physiological and anatomical similarities to humans, are a promising animal model in maternal-offspring nutrition and host-microbiota interaction [1]. Particularly, Bama mini-pigs might be the most suitable preclinical xenotransplantation model [2]. Furthermore, the Bama mini-pig is a prominent local pig breed in China because of its characteristics of delicious meat, higher roughage tolerance, and good adaptability to the local environment. Nevertheless, the small litter size, slow growth rate, and extensive management methods have resulted in a low growth rate, feed conversion, and lean meat rate of Bama mini-pigs [3]. The economic benefits of the Bama mini-pig breeding industry are relatively low due to the occurrence of diarrhea and various stresses during weaning. Thus, improving the intestinal development and health of weaned offspring through maternal nutrition intervention is particularly important.

Betaine, a trimethyl derivative of glycine, is known to function physiologically as an osmotic stress protection and methyl group donor and also maintains intestinal function [4]. A previous study reported that betaine levels at 0–5% of the rodent diet were nontoxic in subacute and subchronic models [5]. Studies on betaine intake in a human study showed that the resting concentrations of betaine in serum ranged from 20 to 70 μmol/L [6]. In addition, betaine reduces the amount of the second limiting amino acid methionine added to the pig diet, which in turn reduces feeding costs [7]. As a promising anti-oxidant agent, betaine and its metabolites can ameliorate lipid peroxidation and sulfur amino acid metabolism and promote glutathione (GSH) synthesis against oxidative stress to improve the growth of animals [8]. Moreover, betaine ameliorates acute severe ulcerative colitis by inhibiting oxidative stress-induced inflammatory pyroptosis [9]. Several studies reported that betaine supplementation could significantly improve anti-oxidant defense markers, including total anti-oxidant capacity (T-AOC), total superoxide dismutase (T-SOD), GSH peroxidase (GSH-Px), and GSH levels in broilers [10,11]. Betaine could relieve inflammation by decreasing the transcription and synthesis of pro-inflammatory cytokines (i.e., interleukin (IL)-1β, IL-6, tumor necrosis factor (TNF)-α) [12,13]. Moreover, a previous study revealed that betaine also suppressed the LPS-induced activation of the TLR4/MyD88 pathway and enhanced zonula occludens (ZO)-1 and occludin levels in IEC-18 cells [14]. Therefore, betaine supplementation in sows and/or their offspring’s diets may influence the offspring piglet’s health by enhancing various biological pathways. Nevertheless, most studies on betaine in animal husbandry are mainly focused on poultry, and the applications in pig production are mostly concerned with carcass quality and fat deposition [4,15]. The effects of maternal-offspring betaine supplementation on the oxidative and inflammatory balance, intestinal health, and gut microbiota in pigs have not been studied extensively.

Our recent study reported that maternal betaine supplementation during gestation and lactation alleviated inflammation and oxidative stress by decreasing the plasma levels of IL-1β, IL-6, TNF-α, and malondialdehyde (MDA) in suckling piglets [16]. However, the effects of betaine supplementation in sows and/or their offspring diets on the offspring piglets’ redox and inflammatory status, colonic barrier function, and colonic microbiota colonization, and the underlying mechanism of betaine regulating the intestinal barrier function still remained unclear. Thus, we hypothesized that betaine supplementation in sows and/or their offspring diets might improve the weaned piglets’ redox status and immune and anti-inflammatory levels, enhance the colonic barrier function, and alter the gut microbiota community. Therefore, the present study explored the impacts of dietary betaine supplementation in sows and/or their offspring on weaned offspring piglets’ redox status, immunity, colonic barrier function, and microbiota alteration.

## 2. Materials and Methods

### 2.1. Animals, Treatments, and Diets

A total of 36 Bama mini-sows d 3 of gestation (similar body conditions; during 3–5 parities) and their weaned offspring piglets (28 d of age) were randomly allocated to the following treatments on: (1) sows and their weaned offspring fed the basal diet (control group, Con group), (2) sows fed the basal diet supplemented with 3.50 kg/t betaine, and their weaned offspring fed the basal diet (sows betaine group, SB group), and (3) sows fed the basal diet supplemented with 3.50 kg/t betaine, and their weaned offspring fed the basal diet supplemented with 2.50 kg/t betaine (sow-offspring betaine group, S-OB group). The sows were housed in single gestation crates (2.20 × 0.60 m) from d 3 to d 104 of gestation. On d 105 of gestation, sows were transferred to the farrowing crates (2.20 × 1.80 m) with a heated floor pad for their offspring piglets until weaning. All piglets were weaned at 28 d of age and transferred into the nursery pens. After weaning, two piglets per litter close to the average body weight (BW) of the litter were selected, and four piglets in the same group were merged into one pen, six pens (replicates) per group. Betaine was added in the form of betaine hydrochloride (95% purity) and provided by Sunwin Biotech Shandong Co., Ltd. (Weifang, Shandong, China). The supplemented dose of betaine referred to previous studies and the tolerance of sows and piglets [17,18].

During the trial, sows were fed with pregnant diets from d 1 after mating to d 104 of gestation and fed lactating diets from d 105 of pregnancy to weaning. The offspring piglets were fed with pre-nursery diets from d 0 to d 60 after weaning and fed post-nursery diets from d 61 to d 90 after weaning. The experimental basal diets were formulated to meet the Chinese Local Swine Nutrient Requirements (NY/T 65-2004) [19], and the diet premixes met the requirements recommended by the National Research Council (NRC 2012). The composition and nutrient levels of basal diets for sows and piglets are presented in Supplementary Tables S1 and S2. All animals had access to water ad libitum throughout the trial and were fed twice daily (at 0800 and 1700 h), and the amount of feed was changed with their body condition and stages of pregnancy and lactation. Specifically, Bama mini-sows were fed an increasing amount (i.e., d 1–15, 0.8 kg/d; d 16–30, 1.0 kg/d; d 31–75, 1.20 kg/d; d 76–90, 1.50 kg/d; d 91–105, 2.0 kg/d; 1 week before delivery 1.0 kg/d) of the pregnancy diet, had free access during d 1–3 of lactation, and had 2.40 kg/d of the lactating diet during d 4–21 of lactation.

### 2.2. Samples Collection

On d 30, 60, and 90 after weaning, one piglet with close to the average BW of each pen was selected, and a total of six piglets (*n* = 6 per treatment, half male and half female) were weighed 12 h after the last feeding. Blood samples (10 mL) were collected from the precaval vein into heparinized vacuum tubes, and plasma was obtained by centrifuging at 2500× *g* for 10 min and 4 °C. Plasma samples were stored at −20 °C for the subsequent determination of cytokines and anti-oxidant-related indexes. Colonic contents (10 cm distally to the cecum) were collected and stored at −80 °C for microbial composition analyses. Colonic mucosa samples were rinsed in phosphate buffer saline (pH 7.20; containing KH_2_PO_4_, 15.44 μΜ; NaCl, 1.55 mM; and Na_2_HPO_4_, 27.09 μΜ), and then the mucosa samples were collected using a glass slide, quickly snap-frozen in liquid nitrogen, and stored at –80 °C for further RNA and protein isolation.

### 2.3. Determination of Plasma Immune and Anti-Oxidant Indexes

The plasma levels of IL-2, interferon (IFN)-α, IFN-γ, IgA, IgM, SOD, GSH, GSH-Px, and MDA were analyzed using ELISA assay kits purchased from Jiangsu Meimian Institute (Mei mian, Yancheng, China) as well as the T-AOC assay kit purchased from Nanjing Jiancheng Bioengineering Institute (Nanjing, China) following the instructions of the kits. The absorbance values were read on a Multiscan Spectrum Spectrophotometer (Tecan, Infinite M200 Pro, Männedorf, Switzerland).

### 2.4. Determination of the Relative Protein Abundances

The colonic tissues from the offspring Bama mini-piglets on d 90 after weaning were selected to detect the relative protein abundances. Total proteins were isolated from samples using RIPA lysis buffer (Beyotime, Nantong, China), which contained 1% protease inhibitors and 1% phosphatase inhibitors cocktail. The concentration was measured with the bicinchoninic acid assay kit (Beyotime, Nantong, China). The resolution of protein was determined via sodium dodecyl sulfate-polyacrylamide gel electrophoresis (SDS-PAGE) followed by transferring onto polyvinylidene difluoride (PVDF) membranes. Then, the nonspecific binding was blocked with 5% bovine serum albumin buffer. The membranes were incubated with the primary antibodies against β-actin (#4970, CST, Boston, MA, USA), claudin (#ab211737, Abcam, Cambridge, UK), occludin (#ab216327, Abcam, Cambridge, UK), ZO-1 (#21773-1-AP, Proteintech, Chicago, IL, USA), E-cadherin (#3195, CST, Boston, MA, USA), TLR4 (#14358, CST, Boston, USA), p-NF-κB (#3033, CST, Boston, USA), p-ERK1/2 (#4370, CST, Boston, MA, USA), Nrf2 (#ab62352, Abcam), p-Nrf2 (bs-2013R, Bioss, Beijing, China), and Keap1 (#8047, CST, Boston, MA, USA) at 4 °C overnight. After that, the membranes were washed with Tris-buffered saline (TBS) Tween 20 buffer (TBS plus 0.02% Tween-20) and incubated with a suitable secondary antibody for 1.5 h at room temperature. Finally, the target bands were visualized using the Alpha Imager 2200 software 4.0 (Alpha Innotech Corporation, San Leandro, CA, USA). The protein abundances were normalized against β-actin and analyzed using ImageJ (National Institutes of Health, Bethesda, MD, USA).

### 2.5. Colonic Contents Microbiota Analysis

Total genomic DNA was extracted from 300 mg colonic luminal contents using Mag-Bind^®^ Stool DNA Kit (Omega, Guangzhou, China) following the manufacturer’s instructions. The DNA concentration and purity were confirmed by agarose gel electrophoresis. The V3–V4 region of the 16S rRNA gene was amplified with the forward primer 338F (5′-GCACCTAAYTGGGYDTAAAGNG-3′) and reverse primer 806R (5′-TACNVGGGTATCTAATCC-3′). The amplicon libraries were sequenced on the Illumina MiSeq platform (Illumina, San Diego, CA, USA) with the MiSeq Reagent Kit v3 600 cycles according to the standard protocols by Shanghai Personal Biotechnology Co., Ltd. (Shanghai, China).

Alpha diversity (including Chao1, Simpson, and Shannon) of the operational taxonomic unit (OTU) level was measured using the OTU table and QIIME2 (2019.4) software. The beta diversity analysis was performed to evaluate the structural variation of colonic microbiota communities. Partial least squares discriminant analysis (PLS-DA) was also performed to determine the microbial variation among the three groups. The differences in the relative abundances of colonic microbiota at the phylum and genus levels were analyzed by the Kruskal–Wallis rank-sum test. Linear discriminant analysis (LDA) effect size (LEfSe) was conducted to identify biomarkers of the microbial taxa among the three groups using the default parameters of 2.00 for LDA.

### 2.6. Statistical Analyses

The plasma and colonic immune and anti-oxidant indexes and the relative protein abundances were analyzed by one-way analysis of variance (ANOVA). The comparative analysis among different groups was conducted using Tukey’s test (SPSS 22.0; SPSS Inc., Chicago, IL, USA). All data are presented as means ± standard error of the mean (SEM). The significance value and a trend toward difference were set at *p* < 0.05 and 0.05 ≤ *p* < 0.10, respectively. The differences in alpha diversity indices and the relative microbial abundances were conducted by the Kruskal–Wallis rank-sum test. A corrected *p* value < 0.05 was considered statistically significant. Biomarkers of the microbial taxa were analyzed using the LEfSe (https://huttenhower.sph.harvard.edu/galaxy/, accessed on 19 October 2023), and the graph was plotted with an LDA score > 2.0.

## 3. Results

### 3.1. Levels of Immunoglobulins and Cytokines in Plasma

**Figure 1:**
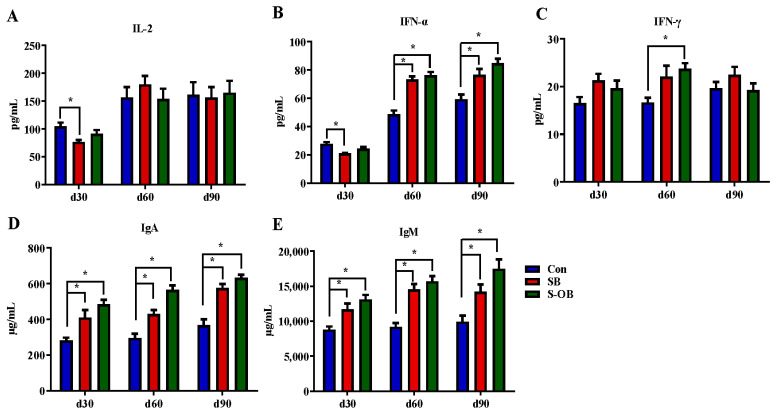
Effects of dietary betaine supplementation in sows and/or their offspring on plasma cytokines (**A**–**C**) and immunoglobulins (**D**,**E**) of offspring Bama mini-pigs on d 30, 60, and 90 after weaning. Data are represented as means ± SEM, *n* = 6 per group. * *p* < 0.05. Con = control group, sows and their weaned offspring fed a basal diet; SB = sows betaine group, sows fed a basal diet supplemented with 3.50 kg/t betaine and their weaned offspring fed a basal diet; S-OB = sow-offspring betaine group, sows fed a basal diet supplemented with 3.50 kg/t betaine and their weaned offspring fed a basal diet supplemented with 2.50 kg/t betaine.

The effects of dietary betaine supplementation in sows and/or their offspring on plasma immunoglobulins and cytokines of offspring Bama mini-pigs are shown in Figure 1. Compared with the Con group, the levels of IgA and IgM in the SB and S-OB groups during d 30–90 after weaning, IFN-α in the SB and S-OB groups on d 60 and d 90 after weaning, and IFN-γ in the S-OB group on d 60 after weaning were higher (*p* < 0.05), while IL-2 and IFN-α levels in the SB group were lower (*p* < 0.05) on d 30 after weaning.

### 3.2. Levels of Oxidative and Anti-Oxidative Indexes in Plasma

**Figure 2:**
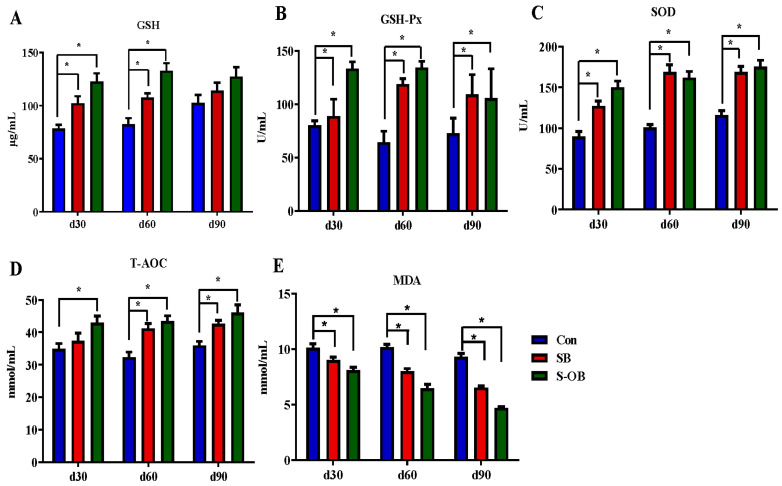
Effects of dietary betaine supplementation in sows and/or their offspring on the plasma levels of anti-oxidation (**A**–**D**) and oxidant indexes (**E**) of offspring Bama mini-pigs on d 30, 60, and 90 after weaning. Data are represented as means ± SEM, *n* = 6 per group, * *p* < 0.05. Con = control group, sows and their weaned offspring fed a basal diet; SB = sows betaine group, sows fed a basal diet supplemented with 3.50 kg/t betaine and their weaned offspring fed a basal diet; S-OB = sow-offspring betaine group, sows fed a basal diet supplemented with 3.50 kg/t betaine and their weaned offspring fed a basal diet supplemented with 2.50 kg/t betaine.

The effects of dietary betaine supplementation in sows and/or their offspring on plasma anti-oxidative and oxidative indexes of offspring Bama mini-pigs are shown in Figure 2. The levels of GSH-Px and SOD were increased (*p* < 0.05), whereas the MDA level was decreased (*p* < 0.05) during the entire trial in the SB and S-OB groups compared with the Con group. The levels of GSH on d 30 and 60 and T-AOC on d 60 and 90 after weaning were increased (*p* < 0.05) in the SB and S-OB groups compared with the Con group. In addition, the level of T-AOC on d 30 after weaning was also increased (*p* < 0.05) in the S-OB group compared with the Con group.

### 3.3. The Relative Abundances of Colonic Tight Junction (TJ) Protein

**Figure 3:**
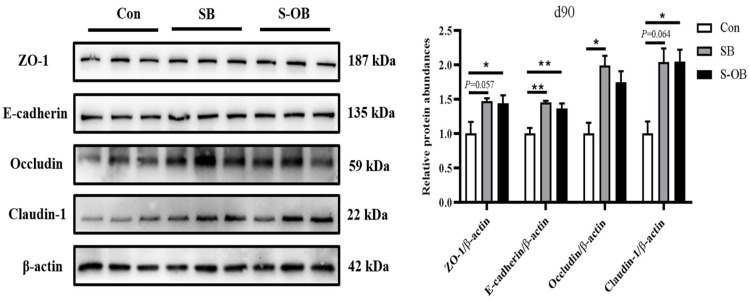
Effects of dietary betaine supplementation in sows and/or their offspring on the relative protein abundances of colonic tight junction in offspring Bama mini-pigs on d 90 after weaning. Data are represented as means ± SEM, *n* = 6 per group, * *p* < 0.05, ** *p* < 0.01. Con = control group, sows and their weaned offspring fed a basal diet; SB = sows betaine group, sows fed a basal diet supplemented with 3.50 kg/t betaine and their weaned offspring fed a basal diet; S-OB = sow-offspring betaine group, sows fed a basal diet supplemented with 3.50 kg/t betaine and their weaned offspring fed a basal diet supplemented with 2.50 kg/t betaine.

The effects of dietary betaine supplementation in sows and/or their offspring on the colonic mucosal TJ proteins of offspring Bama mini-pigs on d 90 after weaning are presented in Figure 3. The relative protein abundances of ZO-1 in the SB (*p* = 0.057) and S-OB (*p* < 0.05) groups, E-cadherin in the SB and S-OB groups (*p* < 0.01), occludin in the SB group (*p* < 0.05), and claudin-1 in the SB (*p* = 0.064) and S-OB groups (*p* < 0.05) were higher than those in the Con group.

### 3.4. Levels of Oxidative and Anti-Oxidative Indexes in Colonic Mucosa

**Figure 4:**
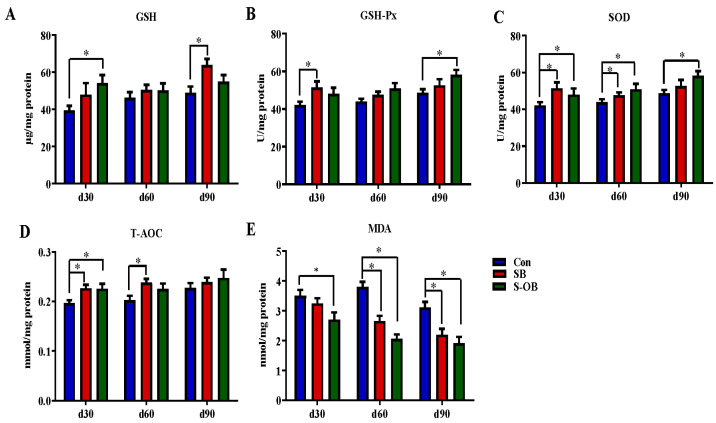
Effects of dietary betaine supplementation in sows and/or their offspring on the levels of anti-oxidation (**A**–**D**) and oxidant indexes (**E**) in the colonic mucosa of offspring Bama mini-pigs on d 30, 60, and 90 after weaning. Data are represented as means ± SEM, *n* = 6 per group, * *p* < 0.05. Con = control group, sows and their weaned offspring fed a basal diet; SB = sows betaine group, sows fed a basal diet supplemented with 3.50 kg/t betaine and their weaned offspring fed a basal diet; S-OB = sow-offspring betaine group, sows fed a basal diet supplemented with 3.50 kg/t betaine and their weaned offspring fed a basal diet supplemented with 2.50 kg/t betaine.

The effects of dietary betaine supplementation in sows and/or their offspring on colonic anti-oxidative and oxidative indexes in the offspring Bama mini-pigs are shown in Figure 4. Compared with the Con group, the levels of T-AOC and SOD in the SB and S-OB groups, GSH in the S-OB group, and GSH-Px in the SB group were increased, while the MDA level in the S-OB group was decreased on d 30 after weaning (*p* < 0.05). On d 60 after weaning, the level of SOD was higher, while MDA was lower in the SB and S-OB groups, and the T-AOC level was higher in the SB group than in the Con group (*p* < 0.05). On d 90 after weaning, the levels of GSH-Px and SOD in the S-OB group and the GSH level in the SB group were higher, while the MDA level in the SB and S-OB groups was lower than in the Con group (*p* < 0.05).

### 3.5. The Relative Abundances of Colonic Nrf2/Keap1 Signaling Pathway

**Figure 5:**
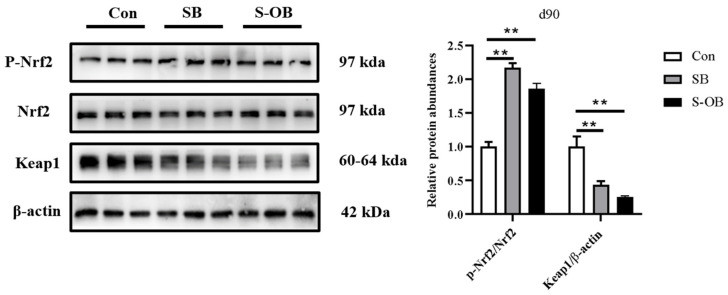
Effects of dietary betaine supplementation in sows and/or their offspring on the colonic mucosal relative protein abundance involved in the Nrf2/Keap1 signaling pathway of Bama mini-piglets on d 90 after weaning. Data are represented as means ± SEM, *n* = 6 per group, ** *p* < 0.01. Con = control group, sows and their weaned offspring fed a basal diet; SB = sows betaine group, sows fed a basal diet supplemented with 3.50 kg/t betaine and their weaned offspring fed a basal diet; S-OB = sow-offspring betaine group, sows fed a basal diet supplemented with 3.50 kg/t betaine and their weaned offspring fed a basal diet supplemented with 2.50 kg/t betaine.

The effects of dietary betaine supplementation in sows and/or their offspring on the colonic mucosal Nrf2/Keap1 signaling pathway in offspring Bama mini-pigs on d 90 after weaning are shown in Figure 5. The colonic relative protein abundances of p-Nrf2/Nrf2 were higher (*p* < 0.01), whereas Keap1 was lower (*p* < 0.01) in the SB and S-OB groups than in the Con group on d 90 after weaning.

### 3.6. The Relative Abundances of Colonic TLR4-NF-κB/MAPK Pathway

**Figure 6:**
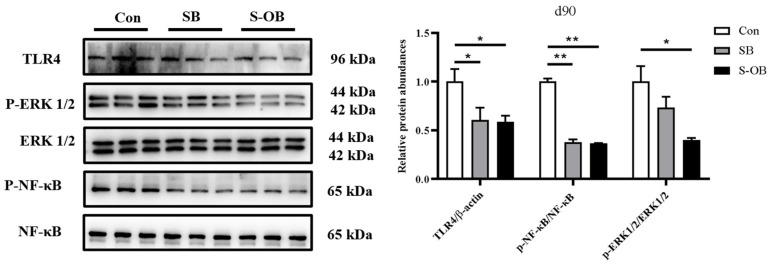
Effects of dietary betaine supplementation in sows and/or their offspring on the colonic relative protein abundances in the TLR4-NF-κB/MAPK pathway of offspring Bama mini-pigs on d 90 after weaning. Data are represented as means ± SEM, *n* = 6 per group, * *p* < 0.05, ** *p* < 0.01. Con = control group, sows and their weaned offspring fed a basal diet; SB = sows betaine group, sows fed a basal diet supplemented with 3.50 kg/t betaine and their weaned offspring fed a basal diet; S-OB = sow-offspring betaine group, sows fed a basal diet supplemented with 3.50 kg/t betaine and their weaned offspring fed a basal diet supplemented with 2.50 kg/t betaine.

The effects of dietary betaine supplementation in sows and/or their offspring on the colonic mucosal TLR4-NF-κB/MAPK pathway of the offspring Bama mini-pigs on d 90 after weaning are shown in Figure 6. Compared with the Con group, the relative protein abundances of colonic TLR4 (*p* < 0.05) and p-NF-κB/NF-κB (*p* < 0.01) were lower in the SB and S-OB groups as well as p-ERK1/2/ERK1/2 (*p* < 0.05) in the S-OB group on d 90 after weaning.

### 3.7. Microbial Composition in Colonic Contents

**Figure 7:**
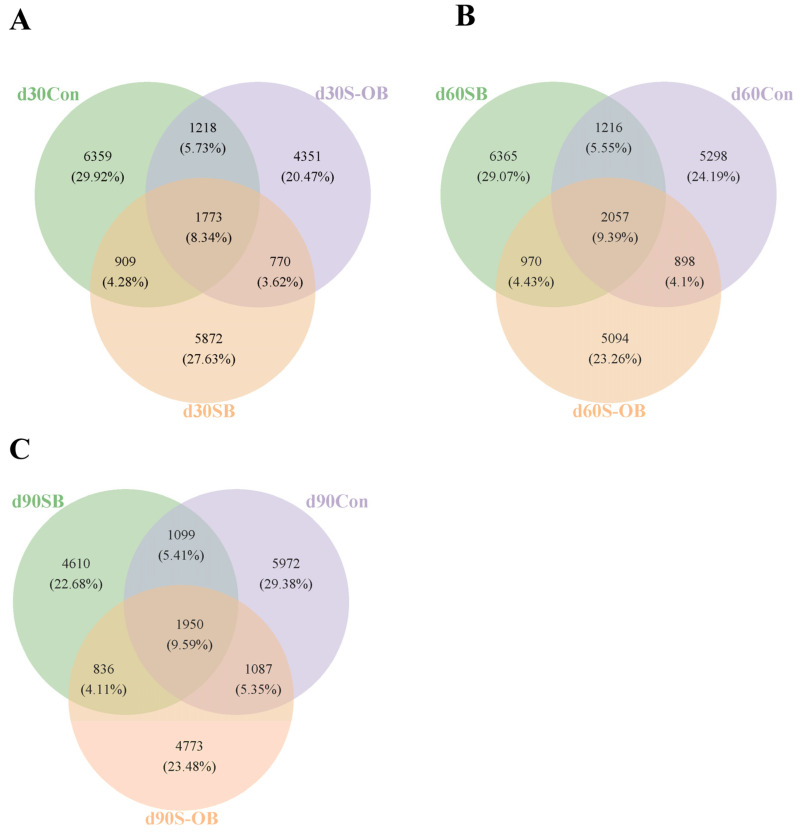
Venn diagrams of core operational taxonomic units (OTUs) in the colonic microbiota of offspring Bama mini-pigs on d 30 (**A**), d 60 (**B**), and d 90 (**C**) after weaning. Different color patterns represent different groups, and the number of overlaps was the shared OTUs among the three groups. *n* = 6 per group. Con = control group, sows and their weaned offspring fed a basal diet; SB = sows betaine group, sows fed a basal diet supplemented with 3.50 kg/t betaine and their weaned offspring fed a basal diet; S-OB = sow-offspring betaine group, sows fed a basal diet supplemented with 3.50 kg/t betaine and their weaned offspring fed a basal diet supplemented with 2.50 kg/t betaine.

The Venn diagram shows OTUs network analysis in the colonic microbiota among the three groups (Figure 7). A total of 21,252, 21,898, and 20,327 microbial OTUs were detected, of which 1773 (8.34%), 2057 (9.39%), and 1950 (9.59%) core microbial OTUs shared on d 30, 60, and 90 after weaning among the three groups, respectively.

**Figure 8:**
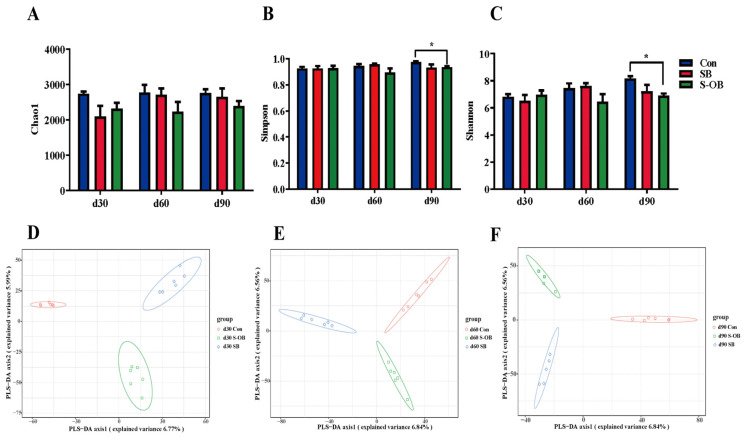
Effects of dietary betaine supplementation on the microbial alpha and beta diversities in the colon of offspring Bama mini-pigs on d 30, 60, and 90 after weaning. Alpha diversity, Chao1 (**A**), Simpson (**B**), and Shannon (**C**) indices of colonic microbiota. Partial least squares discrimination analysis (PLS-DA) in beta diversity of colonic microbiota on d 30 (**D**), 60 (**E**), and 90 (**F**) after weaning. Data are represented as means ± SEM, *n* = 6 per group, * *p* < 0.05. Con = control group, sows and their weaned offspring fed a basal diet; SB = sows betaine group, sows fed a basal diet supplemented with 3.50 kg/t betaine and their weaned offspring fed a basal diet; S-OB = sow-offspring betaine group, sows fed a basal diet supplemented with 3.50 kg/t betaine and their weaned offspring fed a basal diet supplemented with 2.50 kg/t betaine.

The alpha diversity, including the Chao1, Simpson, and Shannon indices, is shown in Figure 8A–C. The Shannon index was lower (*p* < 0.05) in the S-OB group than in the Con group on d 90 after weaning. The Chao1 and Simpson indices showed no significant differences (*p* > 0.05) among the three groups. PLS-DA analysis was performed to distinguish the differences in the microbial community among the three groups. As shown in Figure 8D–F, the beta diversity results showed that there were distinct separations among the three groups on d 30, 60, and 90 after weaning.

### 3.8. Microbiota Community Structure in Colonic Contents

**Figure 9:**
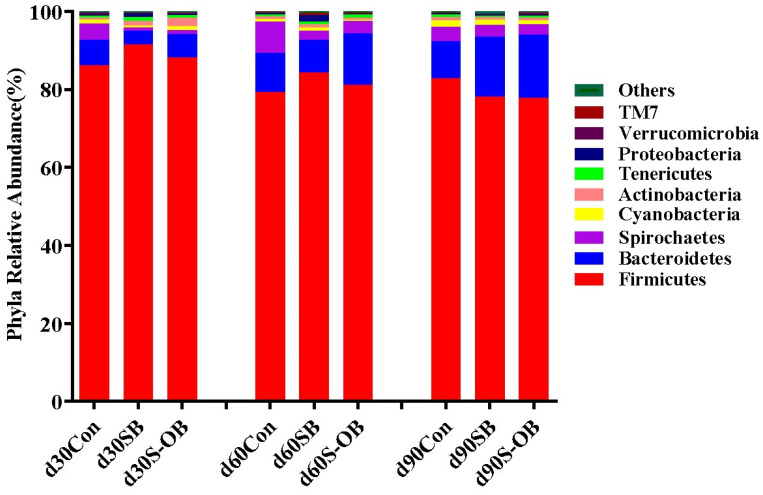
The relative abundance of colonic microbiota at the phylum level of offspring Bama mini-pigs on d 30, 60, and 90 after weaning. The average abundance of phylum > 0.1% was listed. *n* = 6 per group. Con = control group, sows and their weaned offspring fed a basal diet; SB = sows betaine group, sows fed a basal diet supplemented with 3.50 kg/t betaine and their weaned offspring fed a basal diet; S-OB = sow-offspring betaine group, sows fed a basal diet supplemented with 3.50 kg/t betaine and their weaned offspring fed a basal diet supplemented with 2.50 kg/t betaine.

The top nine microbial phyla in the three groups are shown in Figure 9. At the phylum level, the relative abundances of Firmicutes, Bacteroidetes, Spirochaetes, Cyanobacteria, Actinobacteria, Tenericutes, Proteobacteria, Verrucomicrobia, and TM7 were accounted ≥ 0.5%. Firmicutes, Bacteroidetes, and Spirochaetes were the most dominant phyla, accounting for more than 95% of the total colonic microbiota.

**Figure 10:**
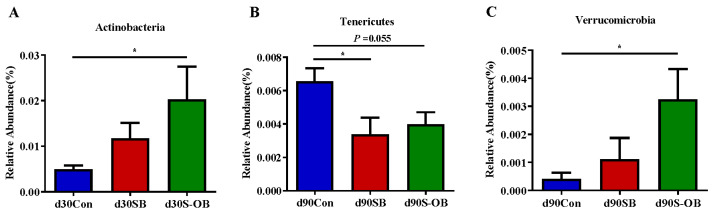
The most abundant colonic microbiota (>0.1%) with significantly different relative abundance at the phylum level (**A**–**C**) of offspring Bama mini-pigs on d 30, 60, and 90 after weaning. Differences in relative abundances were considered significant at *p* < 0.05 using the Kruskal–Wallis rank-sum test. * *p* < 0.05. *n* = 6 per group. Con = control group, sows and their weaned offspring fed a basal diet; SB = sows betaine group, sows fed a basal diet supplemented with 3.50 kg/t betaine and their weaned offspring fed a basal diet; S-OB = sow-offspring betaine group, sows fed a basal diet supplemented with 3.50 kg/t betaine and their weaned offspring fed a basal diet supplemented with 2.50 kg/t betaine.

The most abundant microbial phyla (>0.1%) were analyzed to identify differences in the three age stages (Figure 10). Compared with the Con group, the relative abundances of Actinobacteria on d 30 and Verrucomicrobia on d 90 after weaning were higher (*p* < 0.05) in the S-OB group. The relative abundance of Tenericutes was lower in the SB (*p* < 0.05) and S-OB (*p* = 0.055) groups than in the Con group on d 90 after weaning.

**Figure 11:**
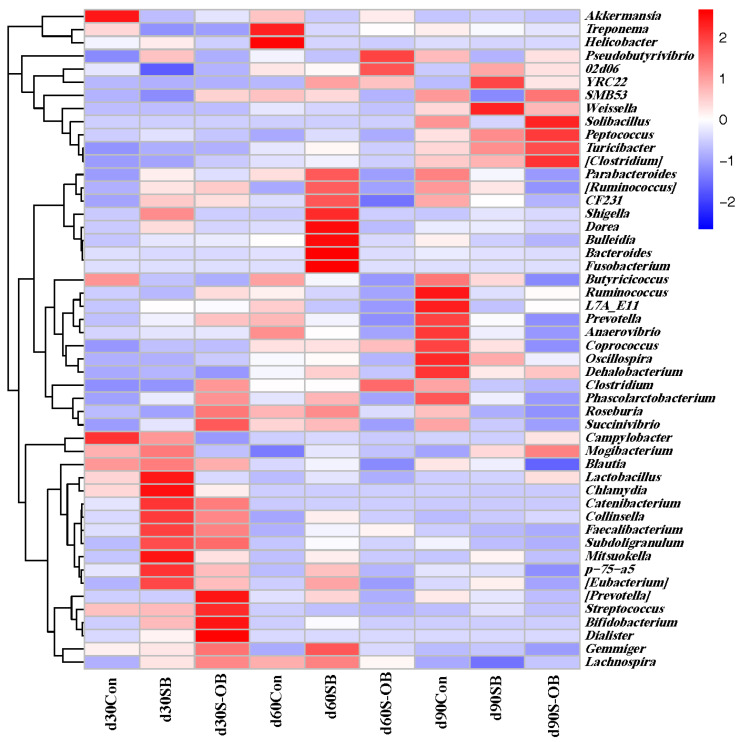
The top 50 bacteria at the genus level of the colonic microbial communities of offspring Bama mini-pigs on d 30, 60, and 90 after weaning. The highest and lowest microbial levels are shown in red and blue, respectively. *n* = 6 per group. Con = control group, sows and their weaned offspring fed a basal diet; SB = sows betaine group, sows fed a basal diet supplemented with 3.50 kg/t betaine and their weaned offspring fed a basal diet; S-OB = sow-offspring betaine group, sows fed a basal diet supplemented with 3.50 kg/t betaine and their weaned offspring fed a basal diet supplemented with 2.50 kg/t betaine.

The top 50 microbial genera in the three groups are shown in Figure 11. The relative abundances of *Blautia*, *Chlamydia*, and *Streptococcus* were higher, and *Turicibacter*, *Clostridium*, *Oscillospira*, and *Dehalobactrium* were lower on d 30 than those on d 60 and d 90 after weaning (*p* < 0.05). The SB group exhibited the highest abundances of *Peptococcus*, *Shigella*, and *[Eubacterium]* and the lowest abundance of *SMB53*. The S-OB group exhibited the highest abundances of *Peptococcus*, *Shigella*, and *[Eubacterium]* while exhibiting the lowest abundances of *Treponema* and *Butyricicoccusy*.

**Figure 12:**
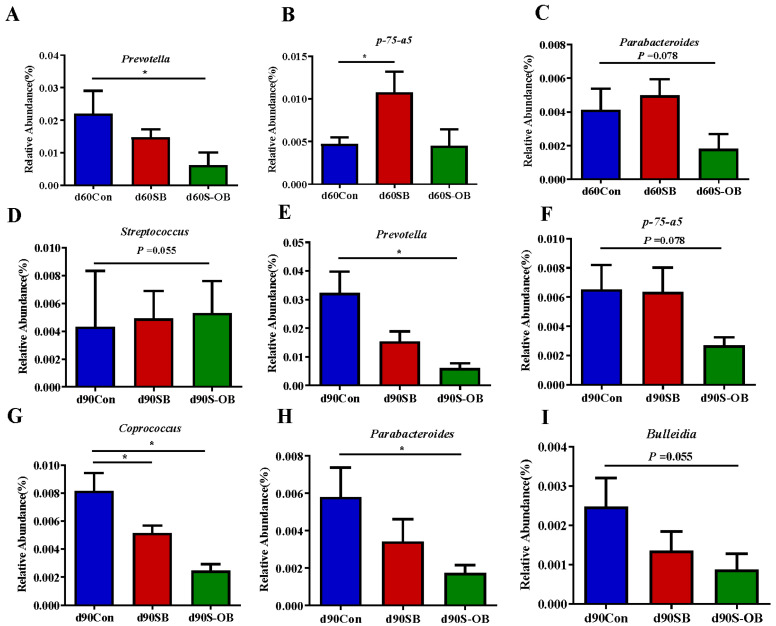
The most abundant colonic bacteria (>0.1%) with significantly different relative abundance at the genus level (**A**–**I**) of offspring Bama mini-pigs on d 30, 60, and 90 after weaning. Differences in relative abundances were considered significant at *p* < 0.05 using the Kruskal–Wallis rank-sum test. * *p* < 0.05. *n* = 6 per group. Con = control group, sows and their weaned offspring fed a basal diet; SB = sows betaine group, sows fed a basal diet supplemented with 3.50 kg/t betaine and their weaned offspring fed a basal diet; S-OB = sow-offspring betaine group, sows fed a basal diet supplemented with 3.50 kg/t betaine and their weaned offspring fed a basal diet supplemented with 2.50 kg/t betaine.

The most abundant microbial genera (>0.1%) were analyzed to identify differences among the three age stages (Figure 12). On d 60 after weaning, the relative abundances of *Prevotella* (*p* < 0.05) and *Parabacteroides* (*p* = 0.078) in the S-OB group and *p-75-α5* (*p* < 0.05) in the SB group were lower than those in the Con group. On d 90 after weaning, the relative abundances of *Prevotella* (*p* < 0.05), *Coprococcus* (*p* < 0.05), *Parabacteroides* (*p* < 0.05), *p-75-α5* (*p* = 0.078), and *Bulleidia* (*p* = 0.055) in the S-OB group as well as *Coprococcus* (*p* < 0.05) in the SB group were lower than those in the Con group.

**Figure 13:**
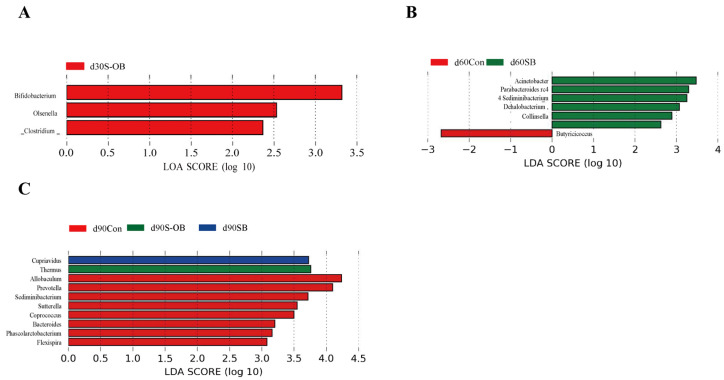
Linear discriminant analysis (LDA) effect size (LEfSe) taxonomic histograms (Kruskal–Wallis rank sum, LDA threshold score > 2.0) in the colonic microbiota of offspring Bama mini-pigs on d 30 (**A**), 60 (**B**), and 90 (**C**) after weaning. *n* = 6 per group. Con = control group, sows and their weaned offspring fed a basal diet; SB = sows betaine group, sows fed a basal diet supplemented with 3.50 kg/t betaine and their weaned offspring fed a basal diet; S-OB = sow-offspring betaine group, sows fed a basal diet supplemented with 3.50 kg/t betaine and their weaned offspring fed a basal diet supplemented with 2.50 kg/t betaine.

The LEfSe analysis was performed to identify the potential biomarker taxa using community-wide responses among the three groups (Kruskal–Wallis rank-sum test). As shown in Figure 13, the *Bifidobacterium*, *Olsenella*, and *Clostridium* abundances were enriched in the S-OB group on d 30 after weaning. On d 60 after weaning, the *Parabacteroides* and *Acinetobacter* abundances were enriched in the SB group. On d 90 after weaning, the *Cupriavidus* abundance was enriched in the SB group, the *Thermus* abundance was enriched in the S-OB group, whereas the *Prevotella* and *Coprococcus* abundances were enriched in the Con group.

## 4. Discussion

Maternal nutrition is a predominant factor in regulating offspring piglets’ redox status, immunity, and gut health [16]. Previous studies have found that dietary betaine improved the anti-oxidant capacity, immune and anti-inflammatory levels, and further promoted the intestinal health of animals [11,12,13,20]. Thus, this study evaluated the effects of betaine supplementation in sows and/or their offspring’s diets on the immune and anti-inflammatory levels and the colonic microbiota community. The findings indicated that betaine supplementation in sows and/or their offspring’s diets influenced the redox status and immune and anti-inflammatory levels of offspring pigs. In addition, dietary betaine supplementation altered the colonic barrier function and microbiota community of the weaned offspring.

Interferons (IFNs) were identified as humoral factors that activate effector cells of the innate and adaptive responses [21]. IFNs, such as IFN-α and IFN-γ, confer an antiviral state upon cells and protect the host from infectious diseases [22]. Furthermore, the immunoglobulins (i.e., IgA and IgM) transmitted from sow’s milk help young animals fend off infections [23]. The present study showed that betaine supplementation in sows and/or their offspring’s diets increased the plasma IgA, IgM, IFN-α, and IFN-γ levels in offspring piglets, suggesting that dietary betaine enhanced the innate immune response in weaned offspring piglets. A previous study also reported that betaine significantly upregulated the IFN-γ mRNA expression, which could promote intestinal permeability [24].

Betaine is a natural compound widely studied as an anti-oxidant in animal diets [4,6]. The mammal possesses several redox defense systems, including SOD, GPX, CAT, and GSH. In the present study, betaine supplementation significantly increased plasma T-AOC, T-SOD, GSH-Px, and GSH levels, whereas it decreased plasma MDA levels in the offspring piglets during the entire trial. Chen et al. [25] reported that betaine supplementation increased the T-SOD level and decreased MDA content in the finishing pigs. Mechanistically, betaine converts homocysteine to methionine, and then methionine is successively converted to S-adenosylmethionine (SAM). Methionine can reduce oxidative injury via chelation and is used by hepatocytes for GSH synthesis [26]. Moreover, SAM is a direct anti-oxidant in the body and can modulate GSH metabolism [27]. Furthermore, the cell experiment showed the hydrophobicity of the three methyl groups and the hydrophilicity of the carboxyl of betaine, and a tight protective membrane was formed around the cells to prevent oxidative stress [28].

The colon is connected with an epithelial monolayer that encompasses an E-cadherin-dependent barrier. In addition, E-cadherin is also considered a tumor suppressor in the colon [29]. Proper epithelial cells are also held together by the apical junctional complex, which includes transmembrane TJ proteins (claudins and occludin) and the cytosolic scaffold proteins (ZO-1) [30]. The present study showed that dietary betaine supplementation up-regulated the colonic protein abundances of E-cadherin, ZO-1, occludin, and claudin on d 90 after weaning, suggesting that betaine can promote the development of intestinal barrier function in weaned offspring piglets. Previously, betaine addition in poultry diets has been found to control intestinal dysfunctional osmoregulatory status, such as ascites and diarrhea [20]. Awad et al. [24] reported that betaine hydrochloride supplementation increased colonic ZO-1, occludin, and claudins abundances, while decreasing IL-1β and IL-13 expressions in broilers. Betaine could improve intestinal functions by enhancing intestinal morphology in rats [31]. Moreover, betaine also enhanced the ZO-1 and occludin levels in the ileal epithelium cell lines [13]. Furthermore, dietary betaine addition improved the relative mRNA expressions of *claudin-1* and *claudin-4* in the intestine of broilers under high ambient temperatures [32]. Therefore, the role of betaine as an osmoprotectant in pig intestines may reduce the maintenance energy needs of Na^+^/K^+^ ion pumps, consequently providing more energy to intestinal epithelial development [33].

Intestinal barrier function is affected by the inflammatory and redox status. The Nrf2/Keap1 oxidative and TLR4-NFκB/MAPK inflammation pathways were detected to explore the above changes in colonic TJ proteins. Under physiological conditions, Nrf2 binds to Keap1 in the cytoplasm. To combat reactive oxygen species (ROS) stress, the Keap1/Nrf2 dissociation prompts the phosphorylation of Nrf2 to translocate into the nucleus and activate the transcription of an anti-oxidant gene. Research evidence proved that an Nrf2 deficiency induced oxidative stress, and inflammation may indirectly impair intestinal barrier function [34]. It has also been reported that betaine supplementation increased the T-AOC, T-SOD, GSH-Px, and GSH levels and decreased MDA content through the up-regulating Nrf2/HO-1/GPX pathway in muscles of broilers [35]. In the present study, dietary betaine supplementation in sows and/or offspring significantly up-regulated protein expression of phosphorylated Nrf2 and down-regulated Keap1 expression in the colon on d 90 after weaning. Activation of the Nrf2 improved the expression of anti-oxidant-related genes and then led to the enhancement of anti-oxidant capacity, which is evidenced by the results of serum and intestinal anti-oxidant capacity indicators.

Pro-inflammatory cytokines, such as TNF-α, disturb intestinal epithelial barrier function and reduce TJ protein expressions and redistribution, leading to increased intestinal permeability [36]. The lipopolysaccharide of Gram-negative bacteria from the external environment activates TLR4 [37], leading to phosphorylated NF-κB to translocate to the nucleus. It also facilitates the transcription of the pro-inflammatory cytokines [38]. TLR4 also activates the downstream ERK1/2 phosphorylation [39]. The present study showed that betaine supplementation in sows and offspring’s diets could decrease the expression of TLR4 and restrain the phosphorylation of ERK1/2 and NF-κB in the colon on d 90 after weaning. Previous studies reported that betaine inhibited the TLR4/MyD88 pathway and up-regulated the mRNA and protein expressions of ZO-1 and occludin in mice with liver injury [13,40]. Meanwhile, betaine also suppresses NF-κB and ERKs activities and their downstream gene (e.g., IL-1β) expression in aged rats and rat endothelial cells [41,42]. Hence, we speculated that dietary betaine could be directly or indirectly absorbed from the feed intake, which might alleviate the intestinal inflammation of piglets and further protect intestinal barrier function.

Nutritional components are also the key factors affecting the structure and abundance of gut community structure. To investigate the effects of dietary betaine on gut microbiota proliferation in offspring Bama mini-pigs, we analyzed the colonic microbiota composition using high-throughput 16S rRNA sequencing. Microbial diversity is considered to improve the stability of microbiota communities [43]. In the present study, the beta-diversity analysis showed that the microbial community structure among the Con, SB, and S-OB groups presented significant separations on d 30, 60, and 90 after weaning, suggesting that betaine supplementation in sows and offspring’s diets altered the colonic microbial community of offspring piglets. The intestine is the harbor of numerous microbial species, belonging predominantly to the phylum Firmicutes or Bacteroidetes [44]. The present study also showed that Firmicutes and Bacteroidetes were the top two dominant phyla in the three groups.

To identify the effects of dietary betaine on the colonic differential bacteria in the offspring Bama mini-pigs, the abundance difference and LEfSe analyses were conducted. At the phylum level, dietary betaine supplementation increased the relative abundances of Actinobacteria and Verrucomicrobia in the colonic contents of offspring piglets. A previous study reported that Verrucobacteria could ameliorate inflammation and is closely related to intestinal health [45]. The most abundant class of the Verrucobacteria phylum, *Akkermansia muciniphila*, as an intestinal symbiont, improves intestinal adaptive immune responses and influences host health [46,47]. Actinobacteria, one of the four major phyla of the gut microbiota, are pivotal in maintaining intestinal homeostasis [48]. Nearly 70% of antibiotics are derived from the extensive secondary metabolism of Actinobacteria [49]. Moreover, the most abundant class of the Actinobacteria phylum, especially *Bifidobacterium*, is widely used as a gut symbiotic probiotic [50]. In addition, dietary betaine supplementation decreased the relative abundance of Tenericutes. Previously, it has been reported that the relative abundance of Tenericutes increased significantly in the inflammatory-aging model, which revealed that the increased Tenericutes abundance was related to inflammation [51]. Therefore, these findings indicate that betaine supplementation in sows and their offspring’s diets might improve the intestinal health of offspring piglets by maintaining gut microbiota homeostasis and promoting anti-inflammatory bacteria colonization.

At the genus level, *Prevotella* and *Bacteroides* are the dominant genera. In the present study, the relative abundance of *Prevotella* was decreased in the SB and S-OB groups on d 60 and d 90 after weaning. Research evidence indicated that *Prevotella* spp., as potential pathogens, are commonly associated with dietary carbohydrates and fiber and linked with intestinal chronic inflammatory conditions [52,53]. A previous study also demonstrated that *Prevotella* colonization deteriorated intestinal inflammation related to colitis [54]. In addition, the relative abundance of *Parabacteroides* was decreased in the SB and S-OB groups on d 60 and d 90 after weaning. As previously mentioned, *P. distasonis* of *Parabacteroides* produced catalases to catabolize ROS, which may exacerbate inflammation [55]. Collectively, these findings indicate that betaine supplementation in sows and their offspring’s diets might reduce gut inflammation by intervening in microbial composition and abundance in weaned pigs.

## 5. Conclusions

**Figure 14:**
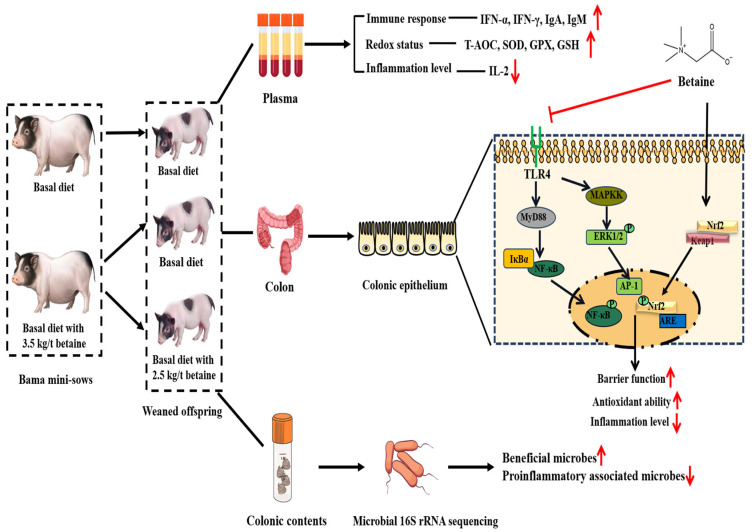
The study design and schematic diagram of effects of betaine supplementation in sows and/or their offspring’s diets on the redox status, immune and inflammatory levels, colonic barrier function, and colonic microbial community of offspring piglets.

In summary, as shown in Figure 14, as betaine supplementation in sows and/or their offspring’s diets could improve offspring piglets’ redox status, immune and anti-inflammatory levels, and enhance the colonic barrier function through activating Nrf2/Keap1 and inhibiting TLR4-NF-κB/MAPK signaling pathways. Moreover, betaine intervened in colonic microbiota composition and decreased proinflammatory-associated microbes. The “sows to piglets” model provides a reference for the maternal-offspring effects of dietary betaine supplementation on gastrointestinal function in humans.

## Data Availability

Please contact the corresponding author if further information is required. The raw datasets of 16S rRNA gene sequencing in colonic microbiota are available in the Science Data Bank under accession number https://doi.org/10.57760/sciencedb.12258 (accessed on 8 September 2023).

## Supplementary Materials

The following supporting information can be downloaded at: https://www.mdpi.com/article/10.3390/antiox12111926/s1, Table S1: Composition and nutrient levels of basal diets for sows (air-dry basis; %); Table S2: Composition and nutrient levels of basal diets for weaned offspring Bama mini-pigs (air-dry basis; %).
