# Supplementary material for: Dietary Betaine Supplementation Enhances Colonic Barrier Function through the Nrf2/Keap1 and TLR4-NF-κB/MAPK Signaling Pathways and Alters Colonic Microbiota in Bama Mini-Pigs
Source: Antioxidants (Basel). 2023 Oct 29;12(11):1926. doi: 10.3390/antiox12111926 (PMC10669150; doi:10.3390/antiox12111926)
Supplement: Supplementary file 1 [file antioxidants-12-01926-s001.zip › antioxidants-2626656-supplementary.pdf]

*antioxidants*

# Dietary Betaine Supplementation Enhances Colonic Barrier Function Through the Nrf2/Keap1 and TLR4-NF-KB/MAPK Signaling Pathways and Alters Colonic Microbiota in Bama Mini-Pigs

**Liang Xiong[1 †], Kai Wang[1, 2 †], Mingtong Song[1], Md. Abul Kalam Azad[1], Qian Zhu[1], Xiangfeng Kong[1 *]**

1 CAS Key Laboratory of Agro-ecological Processes in Subtropical Regions, Hunan Provincial Key Laboratory of Animal Nutritional Physiology and Metabolic Process, Institute of Subtropical Agriculture, Chinese Academy of Sciences, Changsha, Hunan 410125, China
2 Guangdong Provincial Key Laboratory of Silviculture, Protection and Utilization, Guangdong Academy of Forestry, Guangzhou, 510520, China
* Correspondence: nnkxf@isa.ac.cn
† These authors contributed equally to this work.

**Table S1.** Composition and nutrient levels of basal diets for sows (air-dry basis; %)

| Items | Pregnant diet | Lactating diet |
|---|---|---|
| Ingredients | | |
| Corn | 37.50 | 66.00 |
| Soybean meal | 9.50 | 25.00 |
| Wheat bran | 14.00 | 5.00 |
| Barley | 25.00 | |
| Soybean hull | 10.00 | |
| Pregnant premix[1] | 4.00 | |
| Lactating premix[2] | | 4.00 |
| Total | 100.00 | 100.00 |
| Nutrient levels[3)] | | |
| Digestible energy (MJ/Kg) | 12.55 | 13.87 |
| Crude protein | 12.82 | 16.30 |
| Crude fiber | 4.56 | 2.87 |
| SID[4)] Lys | 0.48 | 0.75 |
| SID Met+ Cys | 0.43 | 0.51 |
| SID Thr | 0.37 | 0.53 |
| SID Trp | 0.13 | 0.17 |
| Calcium | 0.62 | 0.65 |
| Phosphorus | 0.47 | 0.50 |

[1)] Pregnant premix provided the following per kg of diet: $CaHPO_4{\cdot}2H_2O$ 10 g, NaCl 4 g, $CuSO_4{\cdot}5H_2O$ 80 mg, $FeSO_4{\cdot}H_2O$ 360 mg, $ZnSO_4{\cdot}H_2O$ 240 mg, $MnSO_4{\cdot}H_2O$ 100 mg, $MgSO_4{\cdot}7H_2O$ 1 g, 1% ICl 50 mg, 1% $Na_2SeO_3$ 36 mg, 1% $CoCl_2$ 16 mg, $NaHCO_3$ 1.4 g, VA 10 000 IU, $VD_3$ 1 800 IU, VE 20 mg, $VK_3$ 2.4 mg, $VB_1$ 1.6 mg, $VB_2$ 6 mg, $VB_6$ 1.6 mg, $VB_{12}$ 0.024 mg, folic acid 1.2 mg, nicotinamide 20 mg, pantothenic acid 12 mg, biotin 0.12 mg, ferrous glycinate 100 mg, choline chloride 1g, phytase 200 mg, fruity 80 mg, and limestone 12 g.

[2)] Lactating premix provided the following per kg of the diet: $CaHPO_4{\cdot}2H_2O$ 10 g, NaCl 4 g, $CuSO_4{\cdot}5H_2O$ 80 mg, $FeSO_4{\cdot}H_2O$ 360 mg, $ZnSO_4{\cdot}H_2O$ 240 mg, $MnSO_4{\cdot}H_2O$ 100 mg, 1% ICl 50 mg, 1% $Na_2SeO_3$ 36 mg, 1% $CoCl_2$ 16 mg, $NaHCO_3$ 1.4 g, VA 10 000 IU, $VD_3$ 1 800 IU, VE 20 mg, $VK_3$ 2.4 mg, $VB_1$ 1.6 mg, $VB_2$ 6 mg, $VB_6$ 1.6 mg, $VB_{12}$ 0.024 mg, folic acid 1.2 mg, nicotinamide 20 mg, pantothenic acid 12 mg, biotin 0.12 mg, Lysine 1.5 g, ferrous glycinate 100 mg, choline chloride 1g, phytase 200 mg, fruity 80 mg, limestone 12 g.

[3)] Nutrient levels were calculated values.

[4)] SID: standard ileum digestible.

**Table S2.** Composition and nutrient levels of basal diets for weaned offspring Bama mini-pigs (air-dry basis; %)

| Items | Prophase diet (28–60 d-old) | Anaphase diet (60–90 d-old) |
|---|---|---|
| Ingredients | | |
| Corn | 54.92 | 58.00 |
| Soybean meal | 22.00 | 18.35 |

| | | |
|---|---|---|
| Wheat bran | 10.13 | 11.35 |
| Rice bran | 8.95 | 8.30 |
| Premix[1)] | 4.00 | 4.00 |
| Total | 100.00 | 100.00 |
| Nutrient levels[2)] | | |
| Digestible energy (MJ/kg) | 13.50 | 13.42 |
| Crude protein | 16.13 | 14.90 |
| Calcium | 0.45 | 0.44 |
| Total Phosphorus | 0.49 | 0.49 |
| Lys | 1.40 | 1.30 |
| Met + Cys | 0.69 | 0.66 |
| Thr | 0.78 | 0.74 |

[1)] Premix provided the following per kilogram of diets: enzyme preparation (including phytase, protease, and lipase) 1.2 g, VA 26 000 IU, $VD_3$ 10 000 IU, VE 70 IU, $VK_3$ 10 mg, $VB_1$ 10 mg, $VB_2$ 25 mg, $VB_6$ 10 mg, $VB_{12}$ 0.075 mg, biotin 0.4 mg, folic acid 5 mg, nicotinamide 100 mg, pantothenic 50 mg, choline 1600 mg, flavoring agent 500 mg, edulcorant 300 mg, acidulating agent 5 g, $CuSO_4{\cdot}5H_2O$ 898 mg, $MnSO_4{\cdot}H_2O$ 298 mg, $ZnSO_4{\cdot}H_2O$ 600 mg, $FeSO_4{\cdot}H_2O$ 501 mg, $Ca(IO_3)_2$ 0.9 mg, as $Na_2SeO_3$ 0.7 mg, $CoSO_4{\cdot}H_2O$ 1.2 mg, glucose 2.1 g, antioxidants 0.4 g, anti-mildew agent 1 g, Ca (as $CaHPO_4$ and $CaCO_3$) 3.42 g, and P (as $CaHPO_4$ ) 1.155 g.

[2)] Nutrient levels were calculated values.
